# Dietary Intake and Representativeness of a Diverse College-Attending Population Compared with an Age-Matched US Population

**DOI:** 10.3390/nu13113810

**Published:** 2021-10-26

**Authors:** Ziaul H. Rana, Cara L. Frankenfeld, Lilian de Jonge, Erika J. Kennedy, Jaclyn Bertoldo, Jerome L. Short, Lawrence J. Cheskin

**Affiliations:** 1 Department of Nutrition and Food Studies, George Mason University, Fairfax, VA 22030, USA; zrana@gmu.edu (Z.H.R.); edejonge@gmu.edu (L.d.J.); 2 Department of Global and Community Health, George Mason University, Fairfax, VA 22030, USA; cfranken@gmu.edu (C.L.F.); ekenne12@gmu.edu (E.J.K.); 3 Department of International Health, Johns Hopkins Bloomberg School of Public Health, Baltimore, MD 21205, USA; jackiebertoldo@jhu.edu; 4 Department of Psychology, George Mason University, Fairfax, VA 22030, USA; jshort@gmu.edu

**Keywords:** college, students, generalizability, dietary assessment, FFQ, DHQ, NHANES

## Abstract

Young adults typically gain more dietary autonomy as they start college, though this can also present nutritional challenges; however, research on the generalizability of their dietary intake data is scarce. To address this representativeness concern, we compared food and nutrient intakes reported by college freshmen attending a large, diverse university to an age-matched sample from the National Health and Nutrition Examination Survey (NHANES). We studied 269 students 18–24 years old recruited through the Mason: Health Start Here (HSH) study, a population-based cohort study of college students. Diet was assessed using a diet history questionnaire (DHQ-III) and estimated with food source composition tables. The NHANES sample of 835 adults was the reference dataset. Reported dietary intakes were weighted and compared with national intakes via t-tests. We observed comparable energy, carbohydrate, fat, and protein intakes in both groups; however, the HSH cohort reported a higher density intake of most micronutrients than the NHANES sample. Differences between these samples in intake, mode of dietary assessment administration, and reactivity may help explain the differences detected. These results demonstrate that when appropriately contextualized in terms of methodology and potential sources of bias, single college studies can be useful for understanding nutrition in young adults more broadly.

## 1. Introduction

Young adulthood is a period of increasing independence for the 40% of young adults enrolled in United States (US) colleges. Diet is a health behavior known to shift substantially in young adulthood, and can have considerable long-term health and other impacts. Previous research indicates that significant environmental changes through adulthood, such as leaving the parental home, entering employment, and changing educational status contribute to poorer nutrition [1]. Young adults are particularly susceptible to depression and becoming overweight or obese, among other conditions, in part due to changing and often worsening nutritional intake in the transition period from high school to college [2]. Research indicates that students learn better when well-nourished, and eating healthfully contributes to several positive learning outcomes, including higher grade averages, increased memory retention and alertness, and faster information processing [3]. Assessing this unique population’s dietary intake is an important first step in decreasing risk factors for common nutrition-related diseases and deficiencies during young adulthood and beyond.

Despite the increased attention being paid to the role of health disparities in health and disease, for which nutrition is a key risk factor, there has been little research on the validity of dietary assessments among college students [4,5,6]. A challenge regarding dietary surveillance, however, is obtaining accurate, comprehensive, and representative data [7,8]. With the advent of new technologies, a variety of innovative assessment tools have emerged, including online tools, which are promising for application in large-scale epidemiological studies. In this respect, web-based, self-administered dietary tools, especially food frequency questionnaires, could enable researchers to obtain accurate dietary data on larger samples, while also saving significant resources [9,10,11]. In some large epidemiological cohort studies, food frequency questionnaires (FFQ) have also been incorporated for the repeated assessment of diet and physical activity, since they assess the variability associated with dietary patterns over time [9,12,13]. Several validation studies have reported good agreement between dietary data collected via online tools compared with those collected via traditional methods [14,15].

To add to the literature on young adults’ nutrition, we undertook the current study, which seeks to better understand the generalizability of food and nutrient intakes reported by college freshmen in the Mason: *Health Starts Here* study. This longitudinal cohort study of successive waves of first-time freshmen was launched in 2019 to improve understanding of the natural history and determinants of young adult physical health, mental health, and college completion. The two primary objectives of the current work were to describe the dietary intake of this college population, and to evaluate the representativeness of the dietary intake and nutrient composition of the cohort by comparing it to an age-matched sample from the *National Health and Nutrition Examination Survey* (NHANES).

## 2. Materials and Methods

### 2.1. Study Design

Mason: *Health Starts Here* (HSH) is a longitudinal cohort study of first-time freshmen at George Mason University (Mason); objectives of the study include examining nutrition and physical activity levels during the college years [16]. The overarching goal of HSH is to identify modifiable risk and protective factors and thus inform new interventions to improve health and well-being. Data collection occurs each fall and spring semester, and each cohort is followed for at least four years. Each fall and spring semester, participants are asked to complete questionnaires, and an in-person visit at study initiation in the fall of the first and fourth years. The online questionnaire each fall includes a food frequency questionnaire. This analysis utilizes baseline data collection gathered in the Mason HSH Cohort wave 1 (first-time freshmen aged 18–24, during the fall of 2019).

NHANES data was obtained from the National Center for Health Statistics [17,18]. NHANES has been conducted continuously since 1999. Data from a nationally representative sample of approximately 5,000 persons per year are released in two-year cycles.

### 2.2. HSH Study Population and Data Collection

The study includes first-time freshmen at George Mason University who are 18 to 24 years old and able to read and understand English. First-time freshman is defined as students who are newly embarking on a four-year undergraduate degree. Participants were students from a variety of disciplines from each college, including Health and Human Services, Humanities and Social Sciences, Visual and Performing Arts, Sciences, and Engineering. In the pilot year (academic year 2019–2020), the study was advertised via email to students in selected courses with large numbers of freshmen enrollees, through flyers placed outside of classrooms, brief presentations to select classes and student organizations, and an online recruitment video on the study website. Three hundred and fifty students were recruited from the incoming freshmen population of 3,704. This study includes the results of the 269 participants after applying the following exclusion criteria: incomplete Dietary History Questionnaire (*n* = 53), unphysiologically low (<500 kcal) reported mean daily dietary intake (*n* = 26), or unphysiologically high (>5000 kcal) energy intake (*n* =2).

The Dietary History Questionnaire (DHQ) used was developed by the National Cancer Institute as an epidemiological research tool to assess total adult diet [19]. DHQ is a dietary recall method based on an FFQ. We used Version III of the DHQ since the nutrient and food group databases were compiled from data from the NHANES dietary recall. A food frequency questionnaire is more appropriate for the HSH study since one of the major objectives of this longitudinal study is to assess the degree to which diet patterns are associated with chronic and non-communicable diseases [16]. DHQ-III includes 135 items about foods and beverages, as well as 26 questions about dietary supplements. Some food and beverage items have embedded questions that allow determination of a final assignment of an item in a nutrient and food group database, leading to 263 foods/beverages in the database. The HSH cohort study used the FFQ reflecting consumption over the month preceding survey administration, including portion sizes. Respondents chose their consumption frequency from a number of categories (1 time in the past month, 2-3 times in the past month, 1 time per week, 2 times per week, 3–4 times per week, 5–6 times per week, 1 time per day, 2 or more times per day), then indicated the portion size (e.g., less than 1 cup, 1 to 2 cups, more than 2 cups). The food groups selected for DHQ-III consist of many distinct individual foods recorded on recalls that are subsumed by the line items. This produced a value per portion size for each nutrient or food group with nutrient values adjusted by gender.

### 2.3. NHANES Study Population and Data Collection

To compare with the HSH Cohort, NHANES data from the most recent two cycles available (2015–2016 and 2017–2018) were used (*n* = 19,225 in the total NHANES sample for those two cycles). The following exclusion criteria were then applied in this order (the number excluded at each stage is in parentheses): age less than 18 or greater than 24 (*n* = 17,819); not having completed a high school education (*n* = 401); unreliable dietary intake as classified by NHANES (*n* = 129); unphysiologically low dietary intake of <500 kcal daily (*n* = 12); unphysiologically high energy intake >5,000 kcal daily (*n* = 15); missing occupation information (*n* = 1); missing smoking information (*n* = 1); and, missing anthropometry data (*n* = 12). There were thus 835 individuals from NHANES who were similarly age and education matched to the HSH study.

NHANES assesses dietary intake through two 24-hour recalls from each participant. The first 24-hour recall is conducted in the Mobile Examination Center (MEC) and the second via telephone 3 to 10 days after the first recall. Dietary intake data are provided as individual food files and total nutrient intake files. As not all participants completed the second 24-hour recall, data from the first 24-hour recall was included in this analysis.

### 2.4. Statistical Analysis

Descriptive statistics are presented as means and standard errors for numerical variables, and frequencies and percentages for categorical variables. Two different assessment methods were used in order to more fairly compare nutrient intake across the cohorts, and given that and underreporting has been documented with food frequencies questionnaires [20]. Percentage of total energy intake for macronutrients and nutrient density were calculated. Energy adjustment for macronutrients was performed by calculating the percent of total energy intake from a given nutrient. Nutrient density was calculated for nutrients by dividing the nutrient by energy intake and multiplying by 1000 (expressed as nutrient per 1000 kcal). Percent calorie intake was estimated for prespecified food groups and tabulated by ranked order. Day 1 dietary sample weights were applied to calculations of NHANES dietary data [21]. Analyses were presented for the combined group and stratified by gender. To compare between the study populations (HSH vs. NHANES, one-sample t-tests were performed to test the statistical significance of the differences between these two populations for each feature. Mean value of the NHANES was used as population mean, as in a prior study [22]. Statistical analysis was performed using Stata 16.1 (StataCorp, USA), with statistical significance set at *p* < 0.05.

## 3. Results

A higher proportion of females participated in HSH than in NHANES (Table 1). The majority of the HSH population was 18 years of age, while in NHANES, participants aged 19 and older were the largest group. Non-Hispanic Whites were the racial/ethnic majority in both studies. The majority of participants in both studies had a normal BMI, but there were more NHANES participants with obesity. Participants were primarily nonsmokers in both studies. More than 40% of the students in the HSH study reported using at least one dietary supplement.

The HSH cohort reported significantly lower energy intake per day than NHANES (Table 2). For the majority of the macronutrients, the observed intake and density for the HSH cohort was lower than age-matched participants in NHANES. These include protein, carbohydrate, saturated fat, mono- and poly-unsaturated fats and cholesterol. Carbohydrate and fiber were the only macronutrients with a higher mean density than among the age-matched NHANES participants. The density observed in almost all the micronutrients for the HSH cohort was higher than the average density for NHANES. In comparison to NHANES, the HSH cohort consumed two times the daily density of vitamin A, three times for vitamin K, and nearly six times for vitamin B12. The only nonsignificant differences among micronutrients were for niacin and phosphorus. 

Grain products were the greatest contributor to daily energy intake for both cohorts (Table 3). Fruits were consumed at a significantly higher level in the HSH cohort, with fruits contributing 10.2% of total daily energy intake. Although high in sugar content, the HSH cohort received 3.2% of daily intake from fruit juices and nectars, compared to a significantly smaller 0.6% among NHANES participants. In the HSH cohort, 9.1% of daily energy consumed was from vegetables, versus 7.2% in the NHANES age-matched cohort. The greatest contributor to the vegetable group for both HSH and NHANES was white potatoes and starchy vegetables. The HSH cohort consumed a significantly greater quantity of milk and milk products, with 14.1% of daily energy coming from this group compared to 8.4% in the NHANES cohort. The NHANES participants (22.1%) consumed 1.33x the daily percentage intake of meat, poultry, fish, and mixtures compared to the HSH cohort (15.5%). One-fifth of the NHANES participants’ daily food group intake came from meat. HSH participants used oils with vegetables and sandwiches significantly more than the NHANES participants. Sugars, sweets, and beverages conveyed a large percentage of the total daily energy intake in both groups.

## 4. Discussion

Generalizability is a major concern when it comes to the assessment of dietary intakes of volunteer college students in epidemiologic research [9,15]. As demonstrated in previous work, web-based FFQ tools have been shown to be feasible and versatile for diet data collection in large-scale studies, and can provide high-quality dietary information that requires less effort and cost than traditional methods [15,23,24]. We undertook the present study to gain a better understanding of the generalizability of food and nutrient intakes reported in a prospective cohort study of 18-24-year-old US college freshman from a diverse university, as this may be applied in future large-scale epidemiologic studies. Specifically, we compared the dietary intakes of freshmen in the *Health Starts Here* (HSH) cohort study at George Mason University with those reported by a nationally representative sample of similarly aged adults from the National Health and Nutrition Examination Survey. While NHANES has some limitations as a comparison group due to it having no specific questions about whether adult participants currently attend school, and uses a different dietary assessment tool, NHANES is the most comprehensive assessment of dietary intake in a study population designed to be representative of the US. These methodological differences provide important context for the comparison we performed.

In order to more accurately compare nutrient intake across cohorts, we focused on nutrient density and percent contributions. We observed comparable energy and macronutrient intakes in both groups. However, the HSH cohort reported a higher density intake of almost all micronutrients than the national sample. We also found percent intakes of fruits, vegetables, and milk were higher in the HSH cohort than in NHANES, while intake of grains and meat was lower in the HSH cohort. Different intake, mode of dietary assessment administration, and reactivity between these groups may in part account for these differences.

Regarding total energy and macronutrient intakes, we found lower intakes among HSH cohort participants than the national sample. Such widespread differences in dietary intake could be in part a result of temporal changes in lifestyle. College freshmen often face major challenges and undergo major adjustments in lifestyle when transitioning to a new, unfamiliar environment as they start college [25]. In addition, voluntary restraint of energy intake has become a common practice among college students, especially among women [26]. Further, differences exist among college students in perspectives concerning choice of diet, nutrition knowledge, and beliefs [27]. We observed a higher percent of caloric intake from fruits and vegetables among the HSH college students than the national average. Increased fruit and vegetable intakes were favorably related to dietary fiber, calcium, magnesium, potassium, folate, and vitamins A, C, E, B1, and B6, and inversely related to trans fats and cholesterol [28]. Recently, some studies have revealed that positive changes also are occurring in food consumption, suggesting that college students are becoming aware of the negative implications of faulty eating habits and are altering their nutrient intakes [29]. Nutrition knowledge has been correlated with increased likelihood of meeting dietary guidelines for fruits and vegetables [30]. In addition, when assessing individual food choices, better nutrition knowledge has been linked to healthier choices [31]. Dietary knowledge, beliefs, and behaviors that are developed and exhibited during college may carry over to adulthood and strongly influence future health status. As fresh fruit is often a relatively costly luxury item, individuals eating in a campus buffet dining hall setting may be more inclined to consume it. These observations suggest that intake differences may reflect access differences. Future research should evaluate whether any differences exist in beneficial nutrients, as well as nutrients of concern between students with and without all-you-can-eat environments. 

Compared to NHANES, this cohort of students had a significantly higher density of micronutrient intakes, which may in part be attributable to dietary supplements. US. college students consume more dietary supplements than the general population, and many consume multiple classes of dietary supplements, despite the lack of scientific evidence to back the benefits of supplement use, and the potential for adverse events [32]. Dietary supplement use appears to be an independent choice, and females are more likely to use them than men [32]. The most frequently reported supplements used are multivitamins, followed by vitamin C, calcium, vitamin E, iron, and folate [33]. Dietary supplements are often consumed in the hopes of increasing energy levels and strength, or in an effort to lose weight, followed by illnesses prevention, as well as supplementation of an inadequate diet [32,33]. The apparent overuse of dietary supplements by college students is of particular concern because many habits established in college persist through life [34]. Epidemiological and clinical studies indicate consistent consumption of some dietary supplements may even increase the risk of morbidity and mortality [35,36]. Therefore, healthful eating habits should be communicated to students to foster lifelong eating habits consistent with accepted nutritional guidelines.

A possible reason for the observed differences in dietary intake between HSH participants and the NHANES sample is a combination of biases associated with selection (participation) or reporting (dietary assessment method). Although both HSH and NHANES focused on nutrition, the HSH study likely attracted more health-conscious college students, whereas NHANES is a cross-sectional study, a major component of the national dietary intake monitoring system. The NHANES recruitment strategies also preclude knowledge of participation and refusal rates. While the distribution of demographic characteristics within the two samples, including age and education, was corrected by the statistical weights, the distributions of other demographic characteristics remain divergent. NHANES consists of urban and rural adults of diverse socioeconomic status, while HSH participants are college students residing in urban areas who, while diverse, tend to have a somewhat higher socioeconomic status. Wide dietary disparities have been observed based on socioeconomic status and region of residency [37]. In addition, as both studies used the same food composition table and applied the same exclusion criteria, the difference in intake could be explained by the reporting method (self-report vs. interview). Lower intakes of nutrients of concern in HSH, particularly when measured based on absolute amount, whereas the energy-adjusted intakes were not as different, suggest that methodological differences may be important.

In general, researchers advocate continued collection of self-reported dietary intake. It is an important part of the overall effort to develop better methods of tracking dietary intake at the population level to inform nutrition policies and conduct research on dietary associations with diseases [9,24]. The web-based dietary recall questionnaire is secure with respect to its relationships with true intake of micronutrients, food groups, and portion sizes; this has been demonstrated through numerous validation studies regarding web-based dietary assessment tools [15]. By comparing 24-hour urinary biomarkers with web-based tool estimates, one study found that the tool performed well in estimating protein consumption, potassium and sodium intake [38]. Therefore, in the future, dietary trend monitoring at the group level could be achieved through repeated application of the same tool within large cohorts of people with heterogeneous diets.

This study has some limitations to consider as part of the overall interpretation. Despite the benefits of an FFQ, a limitation of FFQs is underestimation and misreporting. Questionnaires that use portion sizes may be more likely to underestimate nutrient intakes [15]. In addition, young students may be less motivated and cooperative in recording dietary intakes, leading to inconsistency in results [39]. In some cases, expectancy may cause reporting bias if the students believe that the foods they eat do not adhere to societal norms or guidelines. Another limitation is that most participants resided in urban areas and, on average. had a higher socioeconomic status. Thus, the results may not be generalizable to students living in rural areas or those with a low level of income. As the family income questions in HSH do not align precisely with those in NHANES, it was not possible to compare those groups across the two studies. Finally, although the method is usually standardized, there remain subjective errors in self-reporting processes, changes in nutritional intake over time, and shortcomings of nutritional databases used to calculate intake. 

Nevertheless, the study has several strengths. Despite some disadvantages, cross-sectional studies with sufficient sample size to generate statistical power can be used to assess trends in nutrient intakes and yield useful information [40]. Additionally, this approach is often the only way to obtain information regarding the possible relationship between nutrients and health, and generate hypotheses for future testing [41]. Thus, with a large number of college students participating, this study provides useful information about the dietary intakes of a diverse, representative group of US college students. This diet intake estimation also included the effect of any reported dietary supplements taken. It also provides an updated estimate of nutrient intakes for young adults based on the most recent data on national dietary intake data. 

## 5. Conclusions

We observed that college students in this sample had lower energy intakes, which is likely explained by differences in dietary assessment methodology. However, when controlling for energy intake difference by evaluating nutrient density, the college students in this population consumed higher amounts of most micronutrients. This observation suggests that there may be something about the student experience that contributes to higher micronutrient intake. This corresponds to their higher intake of fruits and vegetables, as well as their consumption of dietary supplements. These factors may be relevant for considering ways to improve micronutrient intake in other populations. The broader utility of these analyses and interpretation illustrates that when appropriately contextualized in terms of methodology and potential sources of bias, studies of single college populations can be useful for understanding nutrition in college students more widely. 

## Figures and Tables

**Table 1:** Baseline characteristics of the Mason: HSH cohort and NHANES age-matched participants.

	HSH (*n* = 269)	NHANES (*n* = 835)
Sex	Mean ± SE or N and %	Mean ± SE or *n* and %
Male	92 (34.2)	403 (51.9)
Female	177 (65.8)	432 (48.1)
Age, y		
18	232 (88.2)	139 (10.3)
19–24	31 (11.8)	696 (89.7)
Mean body weight, lbs		
Male	160.9 ± 4.1	184.7 ± 3.9
Female	141.2 ± 2.5	160.9 ± 4.5
Race/ethnicity		
Non-Hispanic White	103 (38.3)	250 (52.6)
Non-Hispanic Black	33 (12.3)	190 (14.0)
Non-Hispanic Asian	62 (23.1)	82 (5.0)
Hispanic	37 (13.7)	260 (24.4)
Others	34 (12.6)	53 (3.8)
BMI, kg/m^2^		
Underweight (<18.5)	22 (8.3)	46 (5.1)
Normal (18.5 to <25.0)	162 (61.1)	335 (38.1)
Overweight (25.0 to <30.0)	50 (18.9)	191 (24.9)
Obese (30.0 and higher)	31 (11.7)	263 (31.9)
Employment		
Employed	80 (29.85)	528 (70.2)
Unemployed	188 (70.1)	307 (29.8)
Smoking		
Never *	239 (89.2)	675 (76.0)
Ever	29 (10.8)	160 (24.0)
Dietary Supplement		
Male	25 (27.2)	–
Female	85 (48.0)	–
Currently live on Campus		
Yes	190 (73.6)	–
No	79 (29.4)	–
Household Income		
Not enough to get by	9 (3.3)	–
Just enough to get by	71 (26.6)	–
Only have to worry about money for fun and extras	139 (52.1)	–
Never have to worry about money	48 (18.0)	–

* For NHANES, the percentage is weighted percentage, calculated using population weights. Based on the questions asked in NHANES, never smoking is classified as <100 cigarettes in lifetime. (–) data is not collected in the NHANES in the same way as in HSH.

**Table 2:** Daily mean macronutrient intake as percent energy intake, and macronutrient and micronutrient density per 1000 kcal in HSH and NHANES.

Nutrients	HSH Cohort	NHANES	*p*-Value
Energy (kcal)	1610 ± 52	2100 ± 42	**<0.001**
Macronutrients by % total energy			
Carbohydrate (%)	54.5 ± 0.6	47.0 ± 0.5	**<0.001**
Fat (%)	31.8 ± 0.4	35.7 ± 0.5	**<0.001**
Protein (%)	14.7 ± 0.3	16.0 ± 0.5	**<0.001**
Macronutrient g/1000 kcal of intake			
Carbohydrate (g)	136.3 ± 1.6	117.4 ± 1.2	**<0.001**
Protein (g)	36.7 ± 0.7	40.0 ± 1.1	**<0.001**
Total fat (g)	35.4 ± 0.5	39.7 ± 0.6	**<0.001**
Monounsaturated fat (g)	12.8 ± 0.2	13.3 ± 0.2	**0.021**
Polyunsaturated fat (g)	7.5 ± 0.1	9.3 ± 0.4	**<0.001**
Saturated fat (g)	11.9 ± 0.2	13.2 ± 0.2	**<0.001**
Cholesterol (mg)	135.1 ± 5.4	143.7 ± 4.9	0.114
Fiber (g)	10.3 ± 0.3	7.2 ± 0.2	**<0.001**
Micronutrient density (/1000 kcal)			
Vitamin A (ug)	543 ± 26	282± 16	**<0.001**
Vitamin C (mg)	74.1 ± 3.4	33.1 ± 1.4	**<0.001**
Vitamin D (ug)	4.9 ± 0.4	2.17 ± 0.2	**<0.001**
Vitamin E (mg)	7.1 ± 0.6	4.36 ± 0.3	**<0.001**
Vitamin K (ug)	132 ± 10	47 ± 3	**<0.001**
Thiamin (mg)	1.3 ± 0.3	0.80 ± 0.1	**0.037**
Riboflavin (mg)	1.3 ± 0.1	0.98 ± 0.1	**<0.001**
Niacin (mg)	13.5 ± 0.4	13.4 ± 0.3	0.794
Vitamin B6 (mg)	1.5 ± 0.7	1.1 ± 0.1	**<0.001**
Folate (ug)	284 ± 13	92 ± 2	**<0.001**
Vitamin B12 (ug)	20.1 ± 6.5	2.3 ± 0.1	**<0.001**
Calcium (mg)	610± 15	494 ± 15	**<0.001**
Phosphorous (mg)	669 ± 9	682 ± 18	0.172
Iron (mg)	9.3 ± 1.0	6.7 ± 0.1	**0.008**
Magnesium (mg)	176 ± 4	139 ± 4	**<0.001**
Sodium (mg)	1565 ± 25	1727 ± 15	**<0.001**
Potassium (mg)	1463 ± 25	1138 ± 19	**<0.001**
Copper (mg)	0.8 ± 0.1	0.6 ± 0.1	**<0.001**
Selenium (ug)	52 ± 1	58± 2	**<0.001**

Bolded *p*-value means significant (*p* < 0.05).

**Table 3:** Percent energy from major and sub-major food groups in HSH and NHANES.

Mean ± SE (%en)	HSH	NHANES	*p*-Value
Grain products	29.3 ± 0.9	36.0 ± 1.3	**<0.001**
Grain mixtures, frozen meals, soups	11.3 ± 0.6	16.9 ± 1.2	**<0.001**
Pastas, rice, cooked cereals	5.6 ± 0.4	2.3 ± 0.4	**<0.001**
Crackers, snack products	2.8 ± 0.2	3.4 ± 0.4	**0.037**
Cakes, cookies, pies, pastries, bars	2.5 ± 0.2	4.5 ± 0.4	**<0.001**
Pancakes, waffles, French toast, other grain products	2.5 ± 0.2	0.6 ± 0.1	**<0.001**
Yeast breads, rolls	1.7 ± 0.2	4.6 ± 0.3	**<0.001**
Cereals, not cooked	1.6 ± 0.2	1.9 ± 0.3	0.134
Quick breads	1.0 ± 0.1	2.0 ± 0.3	**<0.001**
Vegetables	9.1 ± 0.5	7.2 ± 0.4	**<0.001**
White potatoes, starchy vegetables	6.8 ± 0.4	5.2 ± 0.4	**<0.001**
Dark-green vegetables	0.9 ± 0.1	0.2 ± 0.04	**<0.001**
Orange vegetables	0.3 ± 0.1	0.2 ± 0.05	**0.077**
Tomatoes, tomato mixtures	0.3 ± 0.1	0.5 ± 0.06	**<0.001**
Other vegetables	0.8 ± 0.1	1.14 ± 0.1	**<0.001**
Fruits	10.2 ± 0.6	3.3 ± 0.3	**<0.001**
Citrus fruits, juices	0.6 ± 0.3	0.9 ± 0.1	**<0.001**
Dried fruits	0.2 ± 0.1	0.03 ± 0.02	**<0.001**
Other fruits	6.2 ± 0.4	1.8 ± 0.2	**<0.001**
Fruit juices and nectars excluding citrus	3.2 ± 0.4	0.6 ± 0.1	**<0.001**
Milk and milk products	14.1 ± 0.7	8.4 ± 0.5	**<0.001**
Milks, milk drinks, yogurts, infant formulas	7.0 ± 0.6	3.5 ± 0.4	**<0.001**
Milk desserts and sauces	3.9 ± 0.3	1.3 ± 0.2	**<0.001**
Creams and cream substitutes	1.4 ± 0.2	0.6 ± 0.1	**<0.001**
Cheeses	1.9 ± 0.2	2.9 ± 0.3	**<0.001**
Meat, poultry, fish, and mixtures	15.5 ± 0.7	22.1 ± 0.9	**<0.001**
Poultry	4.9 ± 0.4	5.1 ± 0.5	0.507
Meat, poultry, fish mixtures	3.8 ± 0.3	11.5 ± 0.9	**<0.001**
Organ meats, frankfurters, sausages, lunchmeats	2.5 ± 0.3	1.8 ± 0.2	**0.001**
Beef	1.5 ± 0.2	1.2 ± 0.2	**0.034**
Pork	0.5 ± 0.1	1.1 ± 0.2	**<0.001**
Fish, shellfish	1.0 ± 0.1	1.0 ± 0.2	0.781
Frozen meals, soups, gravies	1.4 ± 0.1	0.3 ± 0.1	**<0.001**
Eggs	3.4 ± 0.4	2.5 ± 0.3	**0.020**
Eggs	3.1 ± 0.3	0.9 ± 0.1	**<0.001**
Egg substitutes	0.3 ± 0.1	0.03 ± 0.02	**<0.001**
Dry beans, peas, other legumes, nuts, and seeds	2.4 ± 0.2	3.1 ± 0.8	**0.004**
Legumes	1.2 ± 0.2	0.7 ± 0.1	**0.003**
Nuts, nut butters, nut mixtures	1.2 ± 0.2	1.7 ± 0.3	**0.006**
Fats, oils, and salad dressings	2.9 ± 0.2	2.1 ± 0.2	**0.001**
For use with a sandwich or vegetable	1.6 ± 0.2	0.2 ± 0.04	**<0.001**
Fats/Oils	0.2 ± 0.1	0.5 ± 0.1	**<0.001**
Salad dressings	1.1 ± 0.1	1.4 ± 0.1	0.063
Sugars, sweets, and beverages	11.1 ± 0.8	15.2 ± 0.7	**<0.001**
Nonalcoholic beverages	4.8 ± 0.4	7.4 ± 0.5	**<0.001**
Sugars, sweets	3.1 ± 0.4	2.7 ± 0.4	0.348
Noncarbonated water	2.1 ± 0.4	0.03 ± 0.02	**<0.001**
Alcoholic beverages	1.1 ± 0.2	3.3 ± 0.5	**<0.001**
Formulated nutrition beverages, energy drinks, sports drink	2.0 ± 0.3	1.8 ± 0.03	0.467

Bolded *p*-value means significant (*p* < 0.05).

## Data Availability

Datasets are available from the corresponding author on reasonable request.
